# Detection of Emerging and Re-Emerging Pathogens in Surface Waters Close to an Urban Area

**DOI:** 10.3390/ijerph120505505

**Published:** 2015-05-22

**Authors:** Stefania Marcheggiani, Emilo D’Ugo, Camilla Puccinelli, Roberto Giuseppetti, Anna Maria D’Angelo, Claudio Orlando Gualerzi, Roberto Spurio, Linda K. Medlin, Delphine Guillebault, Wilfried Weigel, Karim Helmi, Laura Mancini

**Affiliations:** 1 Environmental, Quality and Fishfarm Unit, Environment & Primary Prevention Department, Istituto Superiore di Sanità, Viale Regina Elena 299, 00161 Rome, Italy; E-Mails: emilio.dugo@iss.it (E.D.); camilla.puccinelli@iss.it (C.P.); roberto.giuseppetti@iss.it (R.G.); annamaria.dangelo@iss.it (A.M.D.); laura.mancini@iss.it (L.M.); 2 Laboratory of Genetics, School of Biosciences and Veterinary Medicine, University of Camerino, Camerino, MC 62032, Italy; E-Mails: claudio.gualerzi@unicam.it (C.O.G.); roberto.spurio@iss.it (R.S.); 3 Microbia Environnement, Observatoire Océanologique, 66650 Banyuls/Mer, France; E-Mails: linda.medlin@microbiaenvironnement.com (L.K.M.); delphine.guillebault@microbiaenvironnement.com (D.G.); 4 UPMC Univ Paris 06, CNRS, Laboratoire de Biodiversité et Biotechnologies Microbiennes (LBBM), Sorbonne Universités, Observatoire Océanologique, F-66650 Banyuls/Mer, France; 5 SCIENION AG Volmerstr., 7b/12489 Berlin, Germany; E-Mail: weigel@scienion.de; 6 Centre de Recherche de Saint Maurice, Immeuble le Dufy, Veolia Recherche et Innovation, 1 Place de Turenne, 94417 St Maurice Cedex, France; E-Mail: karim.helmi@veolia.com

**Keywords:** oligonucleotide microarrays, emerging and re-emerging microorganisms, environmental water sample, q-PCR, concentration of water, urban areas

## Abstract

Current knowledge about the spread of pathogens in aquatic environments is scarce probably because bacteria, viruses, algae and their toxins tend to occur at low concentrations in water, making them very difficult to measure directly. The purpose of this study was the development and validation of tools to detect pathogens in freshwater systems close to an urban area. In order to evaluate anthropogenic impacts on water microbiological quality, a phylogenetic microarray was developed in the context of the EU project µAQUA to detect simultaneously numerous pathogens and applied to samples from two different locations close to an urban area located upstream and downstream of Rome in the Tiber River. Furthermore, human enteric viruses were also detected. Fifty liters of water were collected and concentrated using a hollow-fiber ultrafiltration approach. The resultant concentrate was further size-fractionated through a series of decreasing pore size filters. RNA was extracted from pooled filters and hybridized to the newly designed microarray to detect pathogenic bacteria, protozoa and toxic cyanobacteria. Diatoms as indicators of the water quality status, were also included in the microarray to evaluate water quality. The microarray results gave positive signals for bacteria, diatoms, cyanobacteria and protozoa. Cross validation of the microarray was performed using standard microbiological methods for the bacteria. The presence of oral-fecal transmitted human enteric-viruses were detected using q-PCR. Significant concentrations of *Salmonella*, *Clostridium*, *Campylobacter* and *Staphylococcus* as well as Hepatitis E Virus (HEV), noroviruses GI (NoGGI) and GII (NoGII) and human adenovirus 41 (ADV 41) were found in the Mezzocammino site, whereas lower concentrations of other bacteria and only the ADV41 virus was recovered at the Castel Giubileo site. This study revealed that the pollution level in the Tiber River was considerably higher downstream rather than upstream of Rome and the downstream location was contaminated by emerging and re-emerging pathogens.

## 1. Introduction

Contaminated water can be the source of large disease outbreaks caused by many human pathogens [1]. Chemical and microbiological contaminants can occur in surface waters through runoff from agricultural and zoo technical areas, sewage, industrial discharge and wastewaters coming from urban areas [2,3]. Surface waters can also be contaminated through faeces from infected domestic, wild animals and people and can also be greatly affected by extreme weather events, such as heavy precipitation [4,5,6]. The World Health Organization (WHO) recognizes that access to adequate water supplies is a fundamental human right [7]. Water-borne and water-related diseases are usually caused by enteric pathogens that are basically transmitted by the oral-fecal route [8]. These are transmitted to people by inhalation, contact or ingestion of untreated or inadequately treated water and are among the most serious threats to public health today [9]. The various effects on human health caused by waterborne diseases vary in severity from mild to severe, even fatal gastroenteritis, diarrhea, dysentery, hepatitis and typhoid fever. Several studies have confirmed that water-related diseases remain not only a leading cause of morbidity and mortality worldwide, but also the spectrum of disease is expanding and the incidence of many water-related microbial diseases is increasing [9]. Diarrhea is one of the most common features of waterborne diseases and fecal pollution is one of its primary contributors [10,11]. Most waterborne pathogens are introduced into surface water by human or animal feces and can initiate infection in the gastrointestinal tract following ingestion [12]. 

Assessment of water quality impact on public health is complicated because of anthropogenic development and population growth, which exert diverse pressures on water resources quality, quantity and its access. Wastewater effluents as non-point sources of fecal pollution are broadly distributed in both urban and agricultural areas. In particular, urban development impacts strongly on water quality mainly because of the heavy load of human and animal excreta as a main source of water pollution [13,14]. Although a significant proportion of this immense burden of disease is caused by “classical” water-related pathogens, such as those causing typhoid and cholera, newly recognized pathogens and new strains of established pathogens are being discovered [9,15,16].

In the last decade, emerging infectious diseases caused by newly identified or known microorganisms have increased worldwide [9,15,16]. In light of the above considerations, even if the water appears pristine it must be tested to ensure that it contains no harmful microorganisms. Microbiological indicators, such as *Escherichia coli* and *Enterococci*, are widely used in the monitoring programs for regulatory control and in human health risk assessment [17,18]. These indicators may also point out the presence of other non-bacterial pathogens, such as enteric viruses and parasitic protozoa [19]. 

The importance of water in the transmission of recognized pathogens is being constantly reassessed because scientific, technological and epidemiological progresses provides new tools for the evaluation and the analysis of the ecological status of water bodies [20]. Most pathogenic bacteria, viruses, toxic algae and toxins tend to occur at low concentrations, making them very difficult to measure directly. This means that large volumes of water must be collected and filtered as the primary method for concentration. With this concentration comes the concentration of potential inhibitors that can be a big problem for molecular enzymatic reactions such as PCR [21]. Nowhere the pressures for accurate monitoring are felt strongly than at the interface between water and human health [22]. 

Advanced technologies have opened the door for evaluating and characterizing waterborne pathogens through the simultaneous detection of many pathogens. For instance, microarray technology based on the hybridization of fluorescently labeled cDNA or RNA to species specific oligonucleotides is an approach that is now being explored [23,24,25]. To improve our knowledge of emerging and re-emerging pathogens and to better understand the relationship between the environment and human health, we have developed and validated a tool for the detection of pathogens in fresh water systems using microarray technology within the context of the EU project µAQUA to detect simultaneously many pathogens.

## 2. Experimental Section

**Figure 1:**
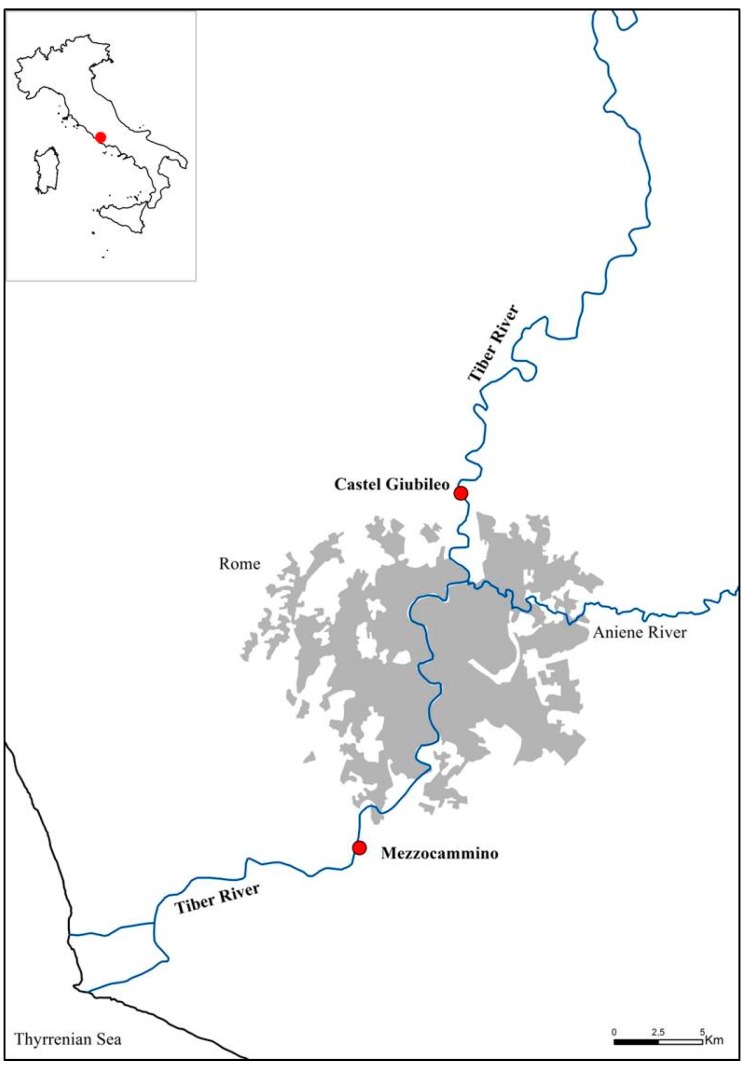
Localization of sampling sites (red dots) and the urbanized region of Rome (gray area).

The Tiber River is one of the largest river systems in Italy, with a catchment area of 17,375 km^2^. The river is 405 km long, and runs through four administrative regions from the Tuscan-Emilian Apennines to the Tyrrhenian Sea. The water volume ranges from 60 m^3^·s^−1^ to 3200 m^3^·s^−1^, with a yearly average of 230 m^3^·s^−1^ [26]. The study area is located in the lower course of the Tiber River close to the urban area of Rome. Two sampling sites were selected on the Tiber River, one upstream (Castel Giubileo-CG: 41°59″14″N; 12°29′39″E) and one located downstream (Mezzocammino-MC: 41°48′25″N; 12°25′05″E) of the city of Rome (Figure 1). The aim was to allow a comparison of harmful microorganisms in surface waters close to an urban area.

### 2.1. Sample Collection

Water samples were collected in May 2012. From each site, water was collected in separate 5-liter and 50-liter sterile carboys and transported and preserved at 4 °C. The fifty liters of water were concentrated to 1 L using a hollow-fiber ultrafiltration approach (HF80S, Fresenius Medical Care, Fresnes, France) and 500 mL of the concentrated eluate was sequentially filtered through the eight different filters of decreasing pore size (20 µm 10 µm, 5 µm, 2 µm, 0.8 µm, 0.45 µm, 0.1 µm and 0.025 µm filters, Millipore, Billerica, MA, USA). Each filter was placed in 1 mL of Tri-reagent (Sigma Aldrich, St Louis, MO, USA), then stored frozen at −80 °C pooled together for RNA extraction. An additional 5 liters were taken to represent an untreated raw sample and submitted to microorganism isolation as described below within 24 h of environmental water sampling.

### 2.2. Isolation of Bacteria

*Campylobacter*, *Salmonella*, *Clostridium* and *Staphylococcus* were isolated from an aliquot of raw water and an equivalent volume of the eluate from the sample collection by membrane filtration [27,28], using selective agar and counting colonies. The volume of water analyzed for the microorganism detection correspond to that required by the directives for evaluation of drinking water quality or surface water quality [17,18]. 

#### 2.2.1. *Staphylococcus* spp.

Raw water (250 mL) and eluate (5 mL) were filtered through a 0.45 µm pore size filter (113 11406 1304073 Sartorius Stedim Biotech, Goettingen, Germany) using a vacuum pump. The filter was placed onto Baird-Parker Agar (BPA, VM 316106 138 Merck KGaA, Darmstadt, Germany) plates using a surface plating method and incubated at 37 ± 1 °C for 48 h [28]. Typical black or greyish-black colonies were gram stained and catalase activity determined. Presence or absence of a clearing or halo on BPA plates was recorded. Bacterial colonies were classified as *Staphylococci* when their appearance on BPA was typical and were Gram-, catalase- and coagulase-positive (Coagulase test Staphytect plus—1110442 Oxoid Ltd., Basingstoke, UK).

#### 2.2.2. *Salmonella* spp.

Raw water (1 L) and eluate (20 mL) were filtered through a 0.45 µm pore size filter (Sartorius Stedim Biotech) using a vacuum pump. Both filters were transferred into 10 mL of Peptone Water (VM299028 127 Merck KGaA) and incubated at 36 ± 1 °C for 24 h. After this pre-enrichment, 100 µL were inoculated into 10 mL of Rappaport-Vassiliadis broth (VM 236766 109 Merck KGaA) and incubated at 42 ± 1 °C for 18–24 h. After incubation broth was streaked on three plates of MacConckey agar selective medium (VM245165 109 Merck KGaA) using a sterile 1 µL inoculating loop and the plates were incubated at 36 ± 1 °C for 18–24 h. Positive and negative controls in selective medium were prepared from each sample. Five percent of the typical colonies, grown on selective medium, have been retraced on TSA agar (524 047 Oxoid Ltd.) and tests were also performed to confirm their biochemical properties (galactosidase, indole, VP) and their serological confirmation by slide agglutination on the same samples [29,30].

#### 2.2.3. *Campylobacter* spp.

Raw water (250 mL) and eluate (5 mL) were filtered through a 0.45 µm pore size filter (Sartorius Stedim Biotech) using a vacuum pump. Filters were enriched in 100 mL of Bolton’s broth (VM 314668 147 Merck KGaA) with antimicrobial supplements (HC074098 Merck KGaA) and 5% (v/v) of lysed horse blood [31]. The control subsamples (microaerobic subsamples) were incubated in anaerobic jars gassed with a microaerobic gas mix (85% N2, 10% CO_2_, 5% O_2_; Airgas, Radnor, PA, USA) using the evacuation-replacement system MAC Smics Jar Gassing System (Microbiology International, Frederick, MD, USA). Samples were incubated at 42 °C for 48 h. After incubation, 0.1 mL of the enriched broth was streaked onto to modified charcoal cefoperazone deoxycholate agar mCCDA (VM301070 134 Merck KGaA). All agar plates were incubated under microaerobic conditions at 42 °C for 48 h. Presumptive *Campylobacter* colonies were observed under phase contrast microscopy (Olympus BX51, Olympus America Inc., Center Valley, PA, USA) for spiral morphology and darting motility. For *Campylobacter* spp., only small, shiny, round, and gray colonies were considered positive.

#### 2.2.4. *Clostridium* spp.

This species was isolated applied protocol described in Marcheggiani *et al.* [32], Briefly, raw water (1 mL) and eluate (20 µL) were analyzed. The samples were heat-shocked at 80–85 °C for 10 min before cultivation to inactivate vegetative bacteria and enhance sporulation. For optimal growth, we adopted the following plating technique: in two different Petri dishes raw water (1 mL) and eluate (20 µL) samples were placed and Sulphite Polymixine Sulphadiazine (SPS) Agar, (8 mL, Oxoid Ltd.) were added and incubated in an anaerobic jar equipped with a manometer and a CO_2_ generator, at 36 ± 1 °C for up to 24 h. Black colonies characteristic of anaerobic bacteria appeared. Colonies were counted directly. Three replicates for each microorganism’s isolation plus positive and negative controls were performed for each sample. After growth, the colonies were counted and results were expressed as colony forming unit x mL (*cfu*/100 mL). 5% of all colonies on each plate were submitted to biochemical, catalase or oxidase tests to confirm their identification.

### 2.3. Virological Analysis

The Hepatitis A Virus (HAV) was provided by the National Institute for Biological Standards and Control (NIBSC, South Mimms, UK): WHO International Standard, WHO First International Standard for HAV RNA Nucleic Acid Amplification (NAT) Assays NIBSC code: 00/560). Hepatitis E Virus (HEV) was provided by Paul Erlich Institut (PEI, Langen, Germany): World Health Organization International Standard for HEV RNA Nucleic Acid Amplification Techniques (NAT)-Based Assays PEI code 6329/10. Norovirus GI and GII (NoGGI, NoGGII) RNA was kindly provided by Dip. SPVSA—Adempimenti comunitari e sanità pubblica, National Institute of Health of Rome (Rome, Italy). Human Adenovirus 41 (ADV 41) and Human Enteroviruses (HE: Poliovirus 1, Echovirus 7, Coxsackievirus), were kindly provided by CRIVIB—Viral Vaccines, National Institute of Health of Rome.

### 2.4. Nucleic Acid Extraction

Viral RNA/DNA extractions from environmental samples were performed using NucliSens magnetic extraction reagents according to the manufacturer’s instructions (Biomerieux, Craponne, France). 

DNA/RNA extractions were performed from a 0.025 µm filter obtained from 500 mL of back-flush concentrated solution (see above). 2 mL of Lysis Buffer (Biomerieux) were used to resuspend the filtrate.

As suggested by manufacturer’s instructions, in order to evaluate the efficiency of extraction, at 1/10 of volume (0.2 mL: aliquot B) of resuspended filtrate (2.0 mL) was added a titled positive control (Poliovirus 1 or other enteric viruses that we’ll not found in the sample) and processed immediately or stored at −80 °C. The sample (1.8 mL sample and aliquot B) was left to incubate for 10 min at room temperature. 50 μL of magnetic silica beads were mixed with the lysis buffer-sample mixture for 10 min and centrifuged at 1500 g for 2 min at room temperature. The mixture buffer-silica-sample was resuspended with wash buffer (WB1, Biomerieux) and transferred to 1.5 mL tube. Using a magnetic rack (DynaMag™-2 Magnet, Life Technologies, Carlsbad, CA, USA), the sample was washed three times (WB1, WB2, WB3 Biomerieux) and the final pellet resuspended in 100 μL of elution buffer (Biomerieux). The mixture was then incubated in a thermomixer (Thermomixer 5436, Eppendorf, Hambourg, Germany) at 1400 rpm 60°C for 5 min and the eluate (100 μL) collected and used for downstream analysis. 200 uL of reference material including HAV, HEV, HE (Human enteroviruses: Poliovirus1, Echovirus7, Coxsackievirus) and ADV41 were extracted as above reported, dissolving the organic fluid (sera or cellular fluid) in 2 mL tube containing Lysis Buffer (Biomerieux). One-two μL out 100 μL of collected eluate (environmental samples and nucleic acid standards) were used to read the nucleic acids contained as well the 260/230 and 260/280 ratios (SPECTROSTAR nano, BMG LABTECH, Ortenberg, Germany). The nucleic acid extractions were analyzed immediately or stored at −80 °C until further analysis.

All environmental samples were subject to analysis of efficiency of extraction (by q-PCR) adding at the aliquot B (see above), a titled Poliovirus1 volume corresponding to 10^5^ genomic equivalents. The value obtained analyzing by q-PCR 10 out 20 µL (eluate of aliquot B) was compared with a standard curve of HE (see below).

### 2.5. One Step Quantitative PCR of Viruses (q-PCR)

All q-PCRs (environmental sample and viral nucleic acids used as standard) were performed in 50 µL of reaction mixture containing 10 µL of sample (RNA/DNA), 20 µL of Go Taq Probe q-PCR master mix, 0.8 µL of Go Script (GoTaq^®^ Probe 1-StepRT-qPCR System, Promega, Madison, WI, USA), 0.45 µL (900 nM) of primers (forward, P3), 0.9 µL (1.8 mM) of primers (reverse, P4) and 0.25 µL (250 nM) of probe (P5) (Table 1), and 17.6 µL of nuclease-free water. 

**Table 1:** Sequences of primers used to detect viruses in water by q-PCR assays (Human Enterovirus: HE; Hepatitis A Virus: HAV; Norovirus GGI: NoGGI; Norovirus GGII: NoGGI; Hepatitis E Virus: HEV; Adenovirus 41: ADV41. Abbreviations: FAM, 6-carboxyfluorescein reporter dye; BBQ650, Black Berry Quencher 650; BHQ1, Black Hole Quencher.

Viruses	Primer/Probe	Sequences (5′–3′)
Hepatitis A Virus [33]	F	HAV P3 (F): TCA CCG CCG TTT GCC TAG-5'
	R	HAV P4 (R): GGA GAG CCC TGG AAG AAA G
	P	HAV P5 (-) (P): CCT GAA CCT GCA GGA ATT AA. FAM-3'BHQ1
Hepatitis E Virus [34]	F	HEV P3(F): GGT GGT TTC TGG GGT GAC AGG GT
	R	HEV P4 (R): AGG GGT TGG TTG GAT GAA
	P	HEV P5 (P): TGA TTC TCA GCC CTT CGC. MGB-6-FAM
Human Enteroviruses [35]	F	PanE P3(F): GGC CCC TGA ATG CGG CTA ATCC
	R	PanE P4(R): GCG ATT GTC ACC ATW AGC AGY CA
	P	PanE P5 (P): CCG ACT ACT TTG GGW GTC CGT GT5. FAM-3'BHQ1
Human Norovirus GI [36]	F	Noro GI P3 (F): CGC TGG ATG CGN TTC CAT
	R	Noro GI P4 (R): CCT TAG ACG CCA TCA TCA TTT AC
	P	Noro GI P5 (P): TGG ACA GGA GAY CGC RAT CT. TEXAS RED-BBQ 650
Human Norovirus GII [36]	F	Noro GII P3 (F): ATG TTC AGR TGG ATG AGR TTC TCW GA
	R	Noro GII P4 (R): TCG ACG CCA TCT TCA TTC ACA
	P	Noro GII P5 (P): AGC ACG TGG GAG GGC GAT CG. HEX-BBQ 650
Human Adenovirus 41	F	ADV41P3(F): GTACTTCAGCCTGGGGAACA
	R	ADV41 P4 (R): GGTCGACTGGCACGAATC
	P	ADV41 P5 (P): AGACAGGTCACAGCGACTGA. FAM-BHQ1

F, forward/sense; R, reverse/antisense; P, probe; FAM, 6-carboxyfluorescein (reporter dye).

The q-PCR program as follows: initial step 45 °C for 900 s to perform the retro-transcription and 95 °C for 120 s for the inactivation of retro-transcriptase (Go Script, Promega, Madison , VL USA) and the activation of DNA polymerase (GoTaq^®^polymerase, Promega) followed by 45 cycles of: 95 °C for 15 s (denaturation) and 60 °C for 60 s (annealing and extension).

Each tube containing the reaction mixture was divided into two aliquots and subjected to thermal cycler conditions (LightCycler Nano, Roche Diagnostics , Mannheim, Germany). To determine the RT-q-PCR limit of detection and standard curve, tenfold serial dilutions of RNA or DNA (for each virus) with quantities ranging from 10^7^ to 0.1 genomic equivalents, were run under the same conditions. Two wells were used for each standards and sample. To obtain quantitative data on the titer of viral copies in each well, the sample extracts and standards control were subjected to RT q-PCR simultaneously, followed by analysis using LightCycler Nano Software 1.1 (Roche). The values of viral load obtained from each environmental sample were reported per liter dividing the value by 2.5.

All nucleic acids obtained from environmental samples, were subject to inhibition analysis by diluting 10 µL of eluate (aliquot B): 10 and 100 fold. The q-PCR values (genomes equivalent and ΔCq variation between dilutions) were compared with the standard curve of HE (see below). Q-PCRs standard curves of HAV, HEV, HE, NoGGI, NoGGI and ADV41 are listed in Table 2:

**Table 2:** Absolute quantification was analyzed using LightCycler Nano Software 1.1, Roche: Cq = K·log10·(q) + I; (Cq: quantitative cycle; K: curve slope; log10·(q): logarithm of quantity; I: axial intercept; A: Amplification factor; Efficiency: E; *R*^2^: coefficient of correlation).

Viruses	Cq	A	E	*R*^2^
HAV	= −3.3 log10·(q) + 40.81	2.01	100.92%	0.997
HEV	= −3.59 log10·(q) + 41.1	1.9	89.91%	0.9932
HE	= −3.34 log10·(q) + 38.44	1.991	99.25%;	0.9926
NoGGI	= −3.49 log10·(q) + 47.68	1.933	93.43%	0.9928
NoGGII	= −3.39 log10·(q) + 47	1.971	97.24%	0.9979
ADV41	= −3.33 log10·(q) + 41.29	2.0	99.66%;	0.9975

### 2.6. Microarray Design and Description

The protocols used in the EU project µAQUA come from the developments of the MIDTAL project for toxic algae [37]. Briefly probes representing species, genera, classes or phyla of pathogenic bacteria, toxic cyanobacteria, pathogenic protozoa and diatoms as indicator species of water quality were either collected from the literature or newly designed with the ARB program from the ribosomal rRNA genes. All probes were checked *in silico* for the specific recognition of their targets using the nucleotide Basic Local Alignment Search Tool (BLAST, http://blast.ncbi.nlm.nih.gov/Blast.cgi) against the GenBank (http://www.ncbi.nlm.nih.gov/genbank/) and Silva (http://www.arb-silva.de/) databases. Their biophysical properties were analyzed using the Oligonucleotide Properties Calculator software (http://www.basic.northwestern.edu/biotools/oligocalc.html). Positive control probes and higher taxonomic probes targeting kingdom and phylum level came from the MIDTAL microarray [37]. The microarray procedures are commercially available from Microbia Environnement (Banyuls-sur-mer, France). Probes (246) were spotted for this study by Scienion AG (Berlin, Germany) as follows: each microarray slides contained two arrays that contained eight spots for each probe. Hybridizations of each sample were hybridized on different slides. Considering two arrays per sample, each probe is therefore represented by 16 spots. These probes were designed against *Salmonella*, *Shigella*, *Campylobacter*, *E. coli O157:H7*, *Legionella*, *Clostridium botulinum*, *Clostridium perfringens*, *Listeria monocytogenes*, *Staphylococcus aureus*, *Yersinia enterocolitica*, *Vibrio* spp., *Aereomonas* spp., *Bacillus cereus*, *Pseudomonas pp.*, *Microcystis aeruginosa*, *Planktothrix*, *Nodularia spumigena*, *Anabaena* spp., *Aphanizomenon flos-aquae*, *Cylindrospermopsis*, *Cryptosporidium*, *Giardia*, *Entamoeba*, *Naegleria*, *Nitzschia* spp., *Navicula* spp., *Surirella* spp., *Caloneis* spp., *Cyclotella* spp., *Achnanthes* spp., *Cymbella* spp., *Melosira* spp., *Neidium* spp., *Gomphonema* spp., and *Amphora* spp. All the bacterial and cyanobacterial probes were tested for their specificity with RNA extracted from pure or close-relative cultures and environmental cultures for cyanobacteria. They were further tested and characterized by hybridization with field samples for which other means of target identification were used for comparison of microarray efficiency.

### 2.7. RNA Extraction, Labeling and Fragmentation

One mL of the mixture obtained from the pooled filters previously stored in TRI-reagent at −80 °C degree plus an internal extraction quality control (500,000 cells of *Dunaliella tertiolecta*) was processed for RNA extraction using TRlzol^®^ Reagent according to the patented MIDTAL procedure. RNA quality and purity (260/280 and 260/230 ratio) were measured by NanoDrop^®^ Spectrophotometer (Thermo Scientific, Wilmington, DE, USA). The integrity and size distribution of total RNA was checked on an 2100 Bioanalyzer (Agilent,Technologies Inc Santa Clara, CA,, USA). Clean-up was performed using the RNeasy Plus Mini kit (Qiagen, Duesseldorf, Germany). 

One microgram of total RNA extracted of field samples was labeled and purified using a Platinum Bright 647 Infrared Nucleic Acid kit (Leica Biosystem, Nussloch, Germany) according to the manufacturer’s instructions. The degree of labeling (DoL) was determined by measuring concentration and incorporation of the dye using a NanoVue instrument (GE Healthcare, Bucks, UK). Samples with a DoL values between 1.0–3.0 were processed to hybridization. Labeled RNAs were fragmented for 15 min at 85 °C in a salt buffer (100 mM ZnCl_2_ in 100 mM Tris-HCL, pH 7.0) [38]. The reaction was stopped by adding 1/10 volume of 0.5 M EDTA, pH = 8 to the sample. 

### 2.8. Hybridization and Washing

Microarray hybridizations were performed according to protocols published in in Kegel *et al.* [39]. Briefly, labeled field samples (1 µg RNA) were mixed with 2× hybridization buffer containing 3 µL Poly-dA (1 µM) and 10 ng TBP-control made up to a final volume of 30 µL. Poly-dA is added to block the poly-T spacer on the probe and TBP is the TATA box gene fragment added as the positive hybridization control [24]. The labeled RNA was then denatured for 5 min at 95 °C. After denaturation, the samples were placed on ice and 15 µL of KREA block (background blocker from Leica Biosystem, Nussloch, Germany) were added. The hybridization mixture was equally distributed to each array covered with coverslips cleaned with ethanol (LifterSlips, Erie Scientific, VWR International, Radnor, PA, USA)). Slides were placed into an array holder Falcon tube containing a wet Whatman paper and hybridizations were carried out for 1 h at 65 °C. In order to remove un-hybridized RN, the slides were successively washed with three washing steps with increasing buffer stringency under agitation and in the dark to protect the fluorophore. The first buffer (2 × SSC/10 mM EDTA/0.05% SDS) and the second buffer (0.5 × SSC/10 mM EDTA) washings were done at room temperature for 10 min. Finally a third most stringent wash (0.2 × SSC/10 mM EDTA) was performed at 50 °C.

### 2.9. Microarray Scanning 

Fluorescence scanning of slides was performed immediately with the GenePix Pro v5.1 (GenePix 4000B, Microarray Scanner, Molecular Devices, Sunnyvale, CA., USA) microarray scanner with a resolution of 5 μm and an excitation wavelength of 635 nm. The scanner is lasers based. The power of the lasers is be controlled by a pair of high-sensitivity and low-noise photomultiplier tubes (PMT). PMTs are optical components that detect emitted fluorescent light. Increasing the gain of the PMT increases the noise as well as the signal intensity. If the fluorescent signal is saturated at its maximum (65.536 fluorescence intensity), variations between fluorescent signals of the different probes cannot be distinguished.

### 2.10. Data Analysis

The scanned images were analyzed with GenePix analyser software (Molecular Device) to align spots with the corresponding gel files. The signal to noise ratio referring to the probe fluorescence and the background signal, were calculated for each probe by measuring the pixel intensity in the probe defined area minus the background fluorescence corresponding to an average fluorescence around the spots. The hybridization results were analyzed using the hierarchy file and the GPR-Analyzer v1.28 software [40]. The cut-off to consider a positive hit of a set of probes was a normalized signal >0.2 and a signal to noise ratio >2 according the MIDTAL protocol. Positive controls were represented by the TBP and eukaryotic probes (POSITIVE_25_dT, EUK 328, EUK 1209) and the Dunaliella probes (DunGS05_25_dT, DunGS02_25_dT). Negative controls were represented by the Lambda phage probes (GenBank®/EMBL Accession Number J02459).

To compare different hybridization experiments, fluorescent signals were normalized using the internal control DunGS02_25_dT (corresponds to the positive control *Dunaliella tertiolecta*) and the replicates average. 

The results are expressed as mean of the total signal intensity and its standard deviation (SD) for the replicates of each probe.

All microarray results were uploaded to the µAQUA database at http://www.microaqua.eu.

## 3. Results

### 3.1. Microbiological Results

**Figure 2:**
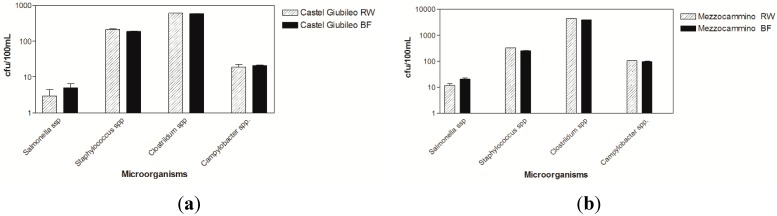
Each column represents a mean value of triplicate measurements of each microorganism, expressed as *cfu*/100 mL, detected at the Castel Giubileo site. (**a**) and Mezzocammino site (**b**). (Striped bar = raw water RW; Black bar = concentrated water BF).

Equivalent volumes of water sample of raw water and eluate were analyzed for isolation of *Campylobacter*, *Salmonella*, *Staphylococcus* and *Clostridium*. Results show that there was no difference between bacteria concentrations (column represents a mean value of three replicates and relative standard deviation) isolated from either raw or eluate water (Figure 2). These results show that the concentration methods used in this study did not affect the detection and viability of microorganisms. However, these results should be further investigated.

Among the Gram-positive bacteria, *Clostridium* spp. were the most prevalent at both sites; followed by *Staphylococcus*. Among Gram-negative bacteria, *Salmonella* was the most prevalent with the highest concentrations detected at the Mezzocammino site followed by *Campylobacter*. Previous studies also recorded the presence in these microorganisms in this area [41].

### 3.2. Virological Results

The results in Table 3 show viral genome detection at both study sites. The nucleic acid amplification detected ADV41 at both sites. Positive results for amplification of HEV, NoGGI and NoGGII were found at the Mezzocammino site. The Castel Giubileo site yielded positive results only for Adenovirus 41. Negative q-PCR results were obtained at both sites for enteric species belonging to the family Picornaviridae, such as HAV and Human enteroviruses (HE), which includes poliovirus, echovirus and Coxsackie virus. All samples were analyzed for extraction and inhibition efficiency as described above (see Experimental Section), using enteric viruses that resulted negative in q-PCR analysis on all environmental samples (*i.e.*, Human enteroviruses, HAV). Poliovirus 1 was chosen as reference material and a q-PCR, previously developed by Oberste *et al.* (Human enterovirus: HE) [42], as method of detection. Ten µL of aliquot B containing genomic equivalents (see Experimental Section) were used to test extraction efficiency and the other 10 µL diluted 10- and 100-fold to evaluate the inhibition. The values obtained were compared to 10-fold serial dilutions of Poliovirus 1 RNA as described in the Experimental Section. No significant reduction of extraction efficiency or evidence of inhibition was observed in any of the analyzed samples.

**Table 3:** Environmental results from the Castel Giubileo and Mezzocammino sites using q-PCR (Hepatitis A Virus: HAV; Norovirus GGI: NoGGI; Norovirus GGII: NoGGII; Human Enterovirus: HE; Hepatitis E Virus: HEV; Adenovirus 41: ADV41). Copies converted to 1 L of water are reported in the respective columns. Negative sample: neg.

Viruses	Castel Giubileo Viral Copies/L	Mezzocammino Viral Copies/L
HAV	neg	neg
NoGGI	neg	10^3^
NoGGII	neg	10^3^
HE	neg	neg
HEV	neg	10^2^
ADV41	10^2^	10^5^

### 3.3. Microarrays

**Figure 3:**
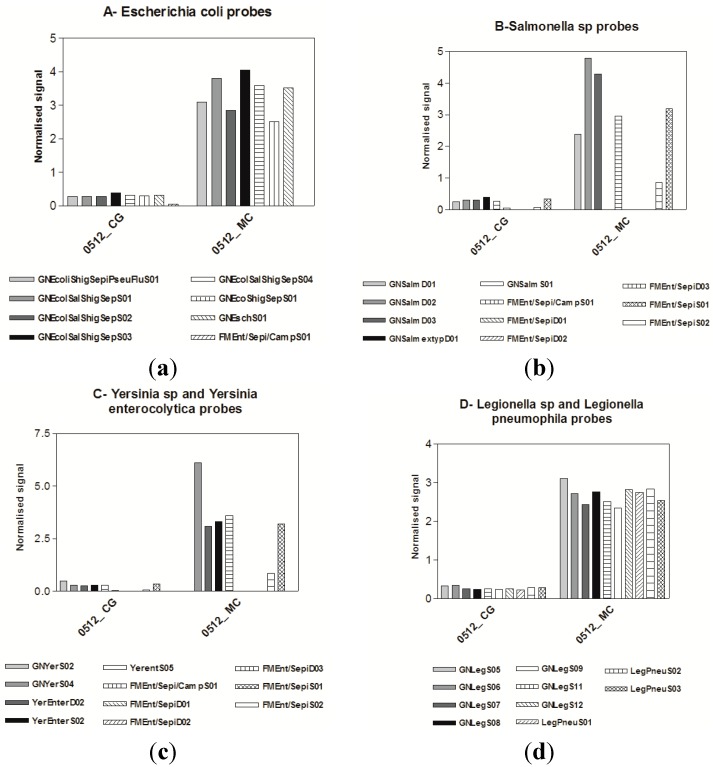

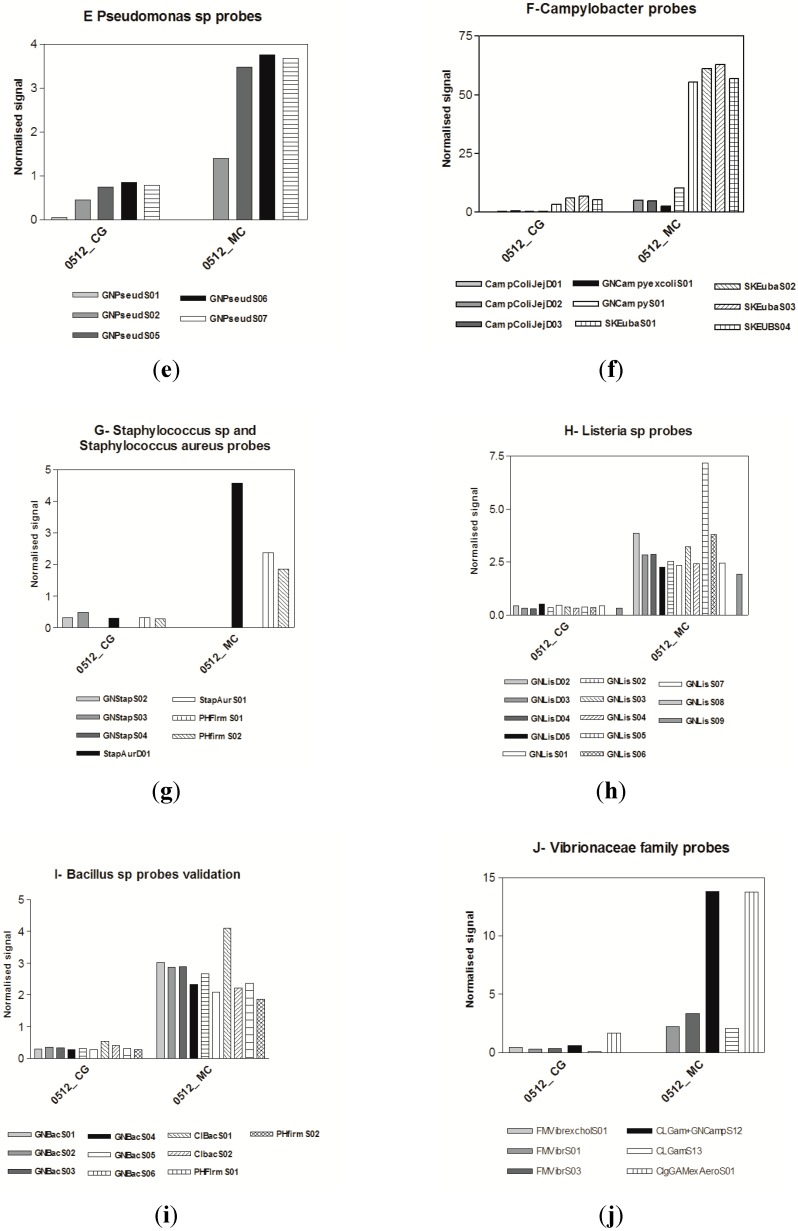
Probes for (**a**) *Escherichia coli*; (**b**) *Salmonella* sp., (**c**) *Yersinia* sp. and *Yersinia enterocolytica*; (**d**) *Legionella* spp. and *Legionella pneumophila*; (**e**) *Pseudomonas* spp.; (**f**) *Campylobacter* spp.; (**g**) *Staphylococcus* and *Staphylococcus aureus*; (**h**) *Listeria* spp.; (**i**) *Bacillus* spp.; (**j**) Family *Vibrionaceae*. Signal intensities of each probe were normalized with *Dunaliella* RNA 12.5 µM probe signal.

**Figure 4:**
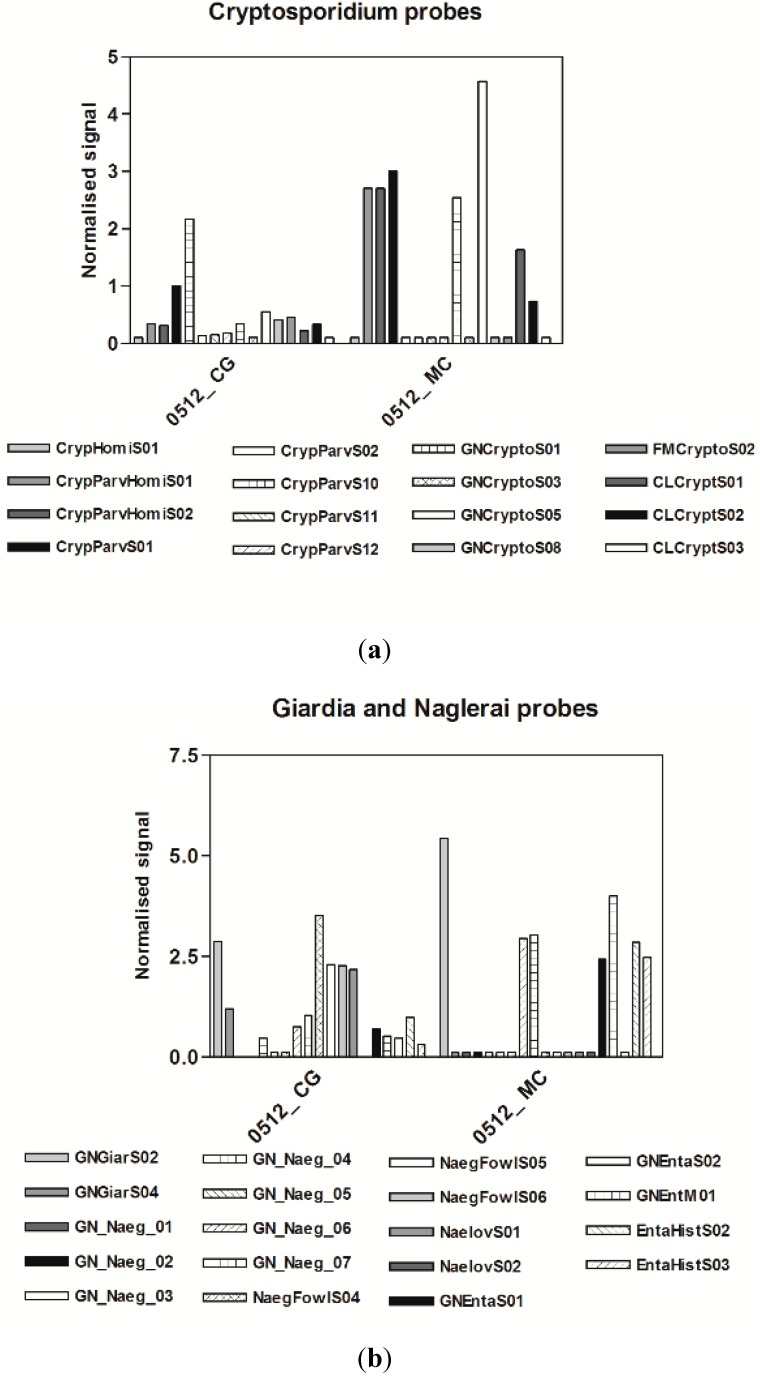
Protozoan probes of the Mezzocammino samples and Castel Giubileo samples. Signal intensities of each probe were normalized with the *Dunaliella* RNA 12.5 µM probe signal.

**Figure 5:**
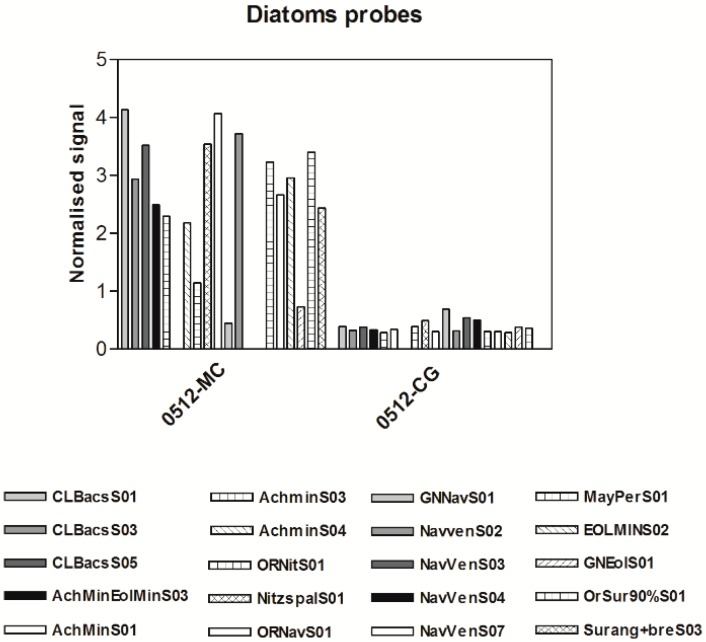
Diatom probes from the Mezzocammino and Castel Giubileo samples. Signal intensities of each probe were normalized with *Dunaliella* RNA 12.5 µM probe signal. *Pseudomonas*, *Bacillus*, *Listeria* *Escherichia* and *Salmonella.* at the genus level and *Staphylococcus aureus* were detected at both sites, with low signal intensities in the Castel Giubileo sample.

Microarray results for target bacteria, protozoa, diatom and cyanobacteria were positive at the two study sites as shown in Figure 3, Figure 4 and Figure 5. Genus and species level probes for *Legionella* spp. and *Legionella pneumophila*, *Yersinia enterocolytica* were highest at the Mezzocammino site. *Campylobacter jejuni/coli* was measured with a higher signal at the Castel Giubileo site. The family Vibrionaceae was measured at both sites with different signal intensities. These bacteria are widespread in brackish and marine waters but they were isolated from a fresh water site not contaminated by faecal waste.

Higher signals were detected for *E. coli*, *Yersenia*, *Salmonella*, *Vibrio* sp., *Legionella*, *Camplylobacter*, and *Listeria* at the Mezzocammino site than at the Castel Giubileo site. The *Clostridium* probes did not detect *Clostridium* RNA very well suggesting either a low cell activity or a probe weakness in recognition of these species.

Similar trends were also observed for protozoa, with higher signal intensities for *Cryptosporidium* and *Giardia* for the Mezzocammino samples than for Castel Giubileo samples. *Naegleria* probes produced significant signals for both sites (Figure 4). The cyanobacterial probes also produced very low signals, which is not surprising because they proliferate in calm waters. Figure 5 shows the diatom species detected in both sites. The signal from the ubiquitous *Achnanthidium minutissimum* [43] was higher at Mezzocammino than for all other probes at the Castel Giubileo site. *Mayamaea atomus var. permitis*, *Eolimna minima*, *Navicula veneta* and *Nitzschia palea*, all species tolerant of nutrients or organic matter, were present at Castel Giubileo, which could indicate eutrophic and mesosaprobic water conditions. A slightly worsening of the ecological status of the waters can be observed from Castel Giubileo to Mezzocamino based on the higher signal intensities of *Navicula veneta* and *Nitzschia palea* as compared to those of *Eolimna minima* and *Mayamaea atomus* var. *permitis* found in Mezzocamino, supporting heavily polluted and polysaprobic conditions [43,44,45].

**Figure 6:**
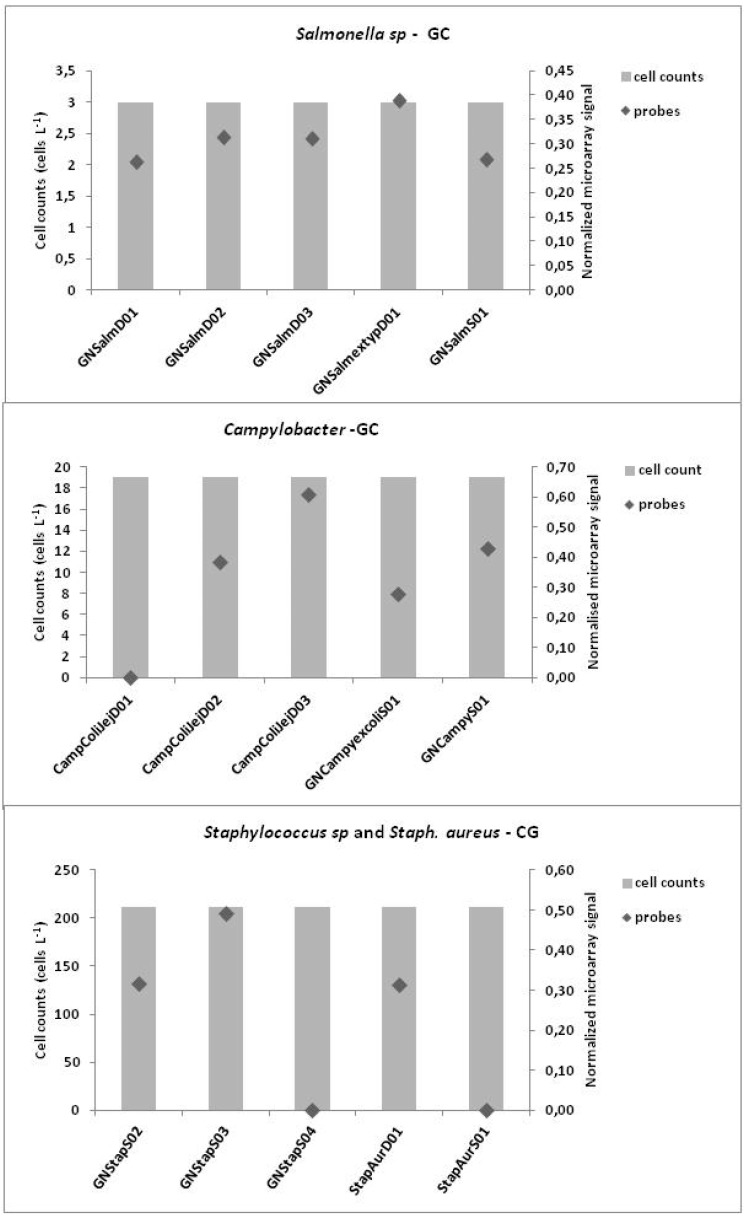
Comparison of concentrations of microorganisms and signal point measurement probes for each probe at the Castel Giubileo site.

**Figure 7:**
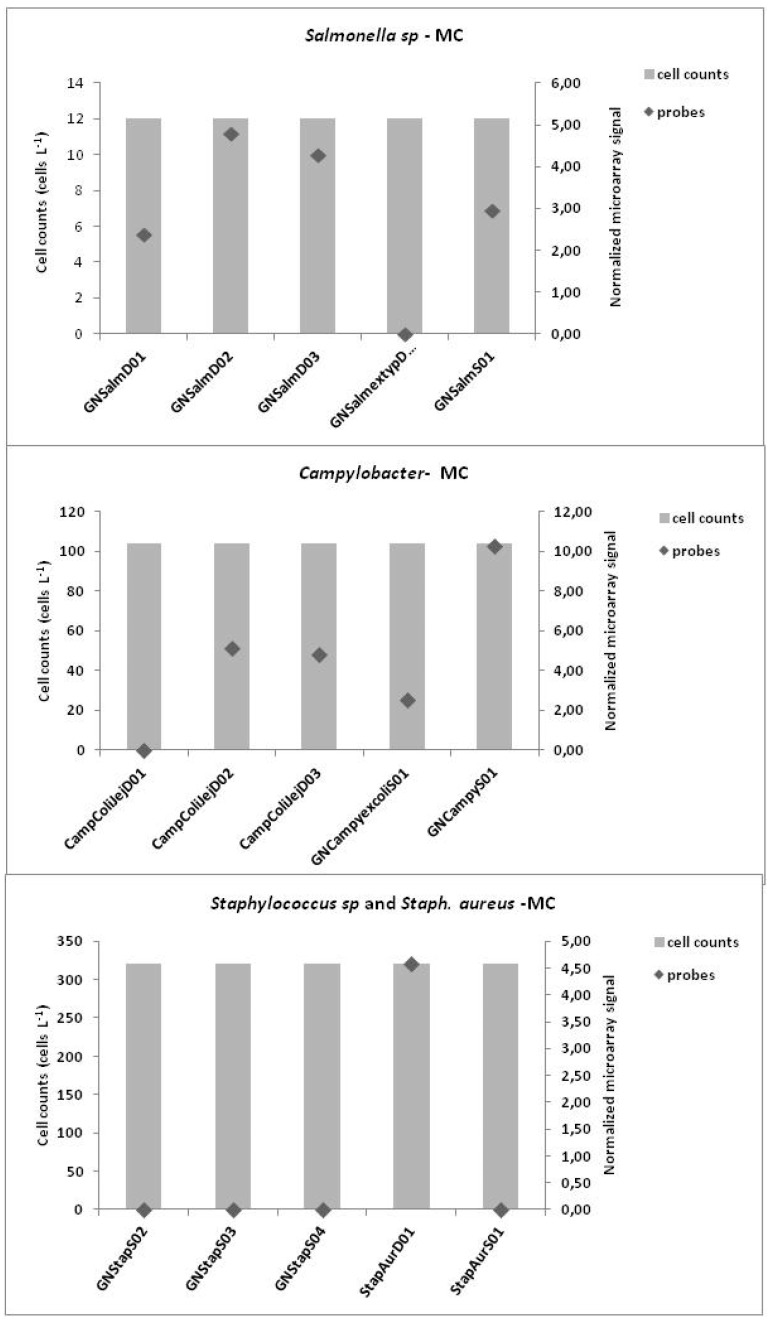
Comparison of concentrations of microorganisms and signal point measurement probes for each probe at the Mezzocammino site.

Finally, the results, expressed in cell concentrations detected at each site obtained by traditional methods for *Campylobacter*, *Salmonella* and *Staphylococcus* were also compared with the results (signal intensity) of probes targeting each microorganism studied present on microarray chip (Figure 6 and Figure 7).

The graphs show the cell concentration of each microorganism detected with traditional methods for each site against each microarray signal probe. The microorganism detections were performed only at the genus level whereas some probes detect the species level. At the Castel Giubileo site, good comparable results were obtained for the CampColiJejD03 probe at the species level and at the genus level for GNSalmextypD01 and GNStapS03. At the Mezzocammino site, the GNSalmD02 and GNCampyS01 probes were comparable at the genus level with the microorganism concentrations.

## 4. Discussion

The monitoring sites are located in the lower course of the river Tiber where many anthropogenic activities are concentrated. Castel Giubileo, located upstream of the city of Rome, is characterized by high agricultural activity. Mezzocammino, located downstream of Rome, is characterized by point and non-source point pollution caused by urbanization and agricultural activities. It also receives waters from the Aniene River, one of the main tributaries inside Rome. Intense industrial activities are found along its course before it joins the Tiber river creating a greater pressure on water quality than those produced by urban and agricultural activities [22]. This catchment area is a typical area with an “Ecosystem Distress Syndrome-EDS” [46] where the inputs exceed the buffering capacity of the river. This situation has more consequences for the ecosystem during in the hot season when low rainfall decreases river discharge. EDS is indicated not only by reduced biodiversity and altered primary and secondary productivity, but also by increased disease prevalence [47]. In general, the loss of balance between the components of the ecosystem together with the effects of climate change, *i.e.*, floods, can favor the growth of pathogenic or potentially pathogenic microorganisms, affecting human and animal health [39].

The results of this study showed that the level of pollution of the Tiber river increases downstream of Rome with the presence of emerging and re-emerging pathogens. Significant concentrations of *Salmonella*, *Clostridium*, *Campylobacter*, *Staphylococcus* bacteria and the presence of HEV, Noroviruses GI and GII and ADV41 viruses were detected at the Mezzocammino site, whereas lower concentrations of bacteria and only ADV41 virus were recovered at Castel Giubileo (Figure 1 and Table 3). The presence of these microorganisms is supported by other studies performed in the same area in previous studies [41]. 

The river water quality decreases dramatically once its tributary, the Aniene River, joins the Tiber River. This is likely a result of the growing exploitation of the Aniene water for agricultural, urban and industrial activities and effects of climate change (*i.e.*, floods) [14,22,48].

Mezzocammino site is located downstream of Rome a few kilometers from the mouth of the Tiber with a pronounced wastewater input from its proximity to urban areas. Furthermore, with temperature increases there is a decrease in river flow, which was typical of our sampling period (May 2012), and which can promote the proliferation of bacteria because growth conditions are optimal [48]. These physical and nutrient conditions likely dictate the presence of the indicator diatoms and bacteria (Figure 5 and Figure 7). Castel Giubileo located upstream of Rome city, showed a lower bacterial signal intensity that corresponded well with a low concentration of bacteria detected with traditional methods (Figure 6 and Figure 7).

The indicator diatom species indicated a bad ecological status at both sites. Another important result of this study is that when pathogens are known and cultivable, the conventional microbiological analysis methods are still an efficient tool to provide information on the infective dose to prevent risks for human health (Figure 1). However, molecular tools are quicker than the current microbiological approaches based on cultures, and they are recommended to set up preventive measures and reduce the human health risks. 

The results of this study contribute to the knowledge of the relationship between environment and health because they gave an idea of what emerging and re-emerging pathogen spread occurs in surface waters (Figure 2 and Table 2). As described in the Introduction section, just *E. coli* and *Enterococci* are commonly used to evaluate water quality for distance bathing and drinking water directives [17,18], whereas the surface Water Framework Directive takes into account biological elements as the primary role, including diatoms, supported by hydromorphological and chemical and physicochemical parameters to evaluate the ecological status [49], so the development of tools to their detection has a fundamental role to prevent the risks caused by direct or indirect consumption of contaminated water. 

## 5. Conclusions

The results of this study showed that the environmental establishment and the spread of emerging and re-emerging pathogens, such as *Staphylococcus* spp., *Campylobacter* spp. and *Salmonella* spp. and viruses in surface waters close to an urban area are constant and remain a serious threat to public health [18]. In an effort to adapt new methods for assessing and managing the risk posed by microbial pollution, we evaluated the utility of oligonucleotide microarrays for bacterial, cyanobacteria, protozoa and diatoms. Specifically, we evaluated the ability of these microarrays to discriminate two environmental samples. Among the main advantages of these methods are their speed and the possibility of using them directly with environmental samples without the need for bacterial cultivation, making them superior to traditional methods. The results of this study contribute to the application of microarrays to the study of environmental problems. This technology constitutes a great opportunity for the simultaneous detection of large number of pathogens and may allow the implementation of preventive measures when it is necessary to monitor the upsurge of emerging pathogens during extreme events such as floods. 
