# Correction: Marcheggiani, S.; *et al.* Detection of Emerging and Re-Emerging Pathogens in Surface Waters Close to an Urban Area. *Int. J. Environ. Res. Public Health* 2015, *12*, 5505–5527

**DOI:** 10.3390/ijerph121013413

**Published:** 2015-10-23

**Authors:** Stefania Marcheggiani, Emilo D’Ugo, Camilla Puccinelli, Roberto Giuseppetti, Anna Maria D’Angelo, Claudio Orlando Gualerzi, Roberto Spurio, Linda K. Medlin, Delphine Guillebault, Julia Baudart-Lenfant, Wilfried Weigel, Karim Helmi, Laura Mancini

**Affiliations:** 1 Environmental, Quality and Fishfarm Unit, Environment & Primary Prevention Department, Istituto Superiore di Sanità, Viale Regina Elena 299, 00161 Rome, Italy; E-Mails: emilio.dugo@iss.it (E.D.); camilla.puccinelli@iss.it (C.P.); roberto.giuseppetti@iss.it (R.G.); annamaria.dangelo@iss.it (A.M.D.); laura.mancini@iss.it (L.M.); 2 Laboratory of Genetics, School of Biosciences and Veterinary Medicine, University of Camerino, Camerino, MC 62032, Italy; E-Mails: claudio.gualerzi@unicam.it (C.O.G.); roberto.spurio@iss.it (R.S.); 3 Microbia Environnement, Observatoire Océanologique, 66650 Banyuls/Mer, France; E-Mails: linda.medlin@microbiaenvironnement.com (L.K.M.); delphine.guillebault@microbiaenvironnement.com (D.G.); 4 UPMC Univ Paris 06, CNRS, Laboratoire de Biodiversité et Biotechnologies Microbiennes (LBBM), Sorbonne Universités, Observatoire Océanologique, F-66650 Banyuls/Mer, France; E-Mail: baudart@obs-banyuls.fr; 5 SCIENION AG Volmerstr., 7b/12489 Berlin, Germany; E-Mail: weigel@scienion.de; 6 Centre de Recherche de Saint Maurice, Immeuble le Dufy, Veolia Recherche et Innovation, 1 Place de Turenne, 94417 St. Maurice Cedex, France; E-Mail: karim.helmi@veolia.com

We wish to make the following changes to the published article [1], agreed upon by all authors: Julia Baudart-Lenfant has been added as co-author. The corrected author list should therefore read: Stefania Marcheggiani, Emilo D’Ugo, Camilla Puccinelli, Roberto Giuseppetti, Anna Maria D’Angelo, Claudio Orlando Gualerzi, Roberto Spurio, Linda K. Medlin, Delphine Guillebault, Julia Baudart-Lenfant, Wilfried Weigel, Karim Helmi and Laura Mancini Author contributions are corrected accordingly:
